# The Prevalence of Reproductive Tract Infections Based on the Syndromic Management Approach Among Ever-Married Rural Women in Kancheepuram District, Tamil Nadu: A Community-Based Cross-Sectional Study

**DOI:** 10.7759/cureus.23314

**Published:** 2022-03-19

**Authors:** Surya Balakrishnan, Archana Carolin, Sudharsan B, Shivasakthimani R

**Affiliations:** 1 Community Medicine, Panimalar Medical College Hospital and Research Institute, Chennai, IND; 2 Community Medicine, Vels Medical College and Hospital, Chennai, IND; 3 Community Medicine, Velammal Medical College Hospital and Research Institute, Madurai, IND

**Keywords:** ever-married females, symptoms of rti, menstrual hygiene, reproductive age group, rti

## Abstract

Introduction

Reproductive tract infections (RTIs) are endemic among developing countries and common among females specifically in the reproductive age group. The sequelae of this lead to infertility. The main reason behind the high prevalence was found to be the lack of awareness about the disease and the stigma toward the disease.

Aims and objectives

This study aims to assess the prevalence of reproductive tract infection based on the syndromic management approach among ever-married rural women in the reproductive age group in the Kancheepuram District.

Methodology

This community-based cross-sectional study was conducted in the rural field practice area of Chettinad Hospital and Research Institute during the period from March 2016 to May 2017. The sample size taken was 330, and the sample size was arrived at by multistage random sampling and population proportion to size. Data were collected using a standardized questionnaire of District Level Household Survey 4 (DLHS-4) on RTI/sexually transmitted infections (STIs). Data were then entered in Microsoft Excel (Microsoft Corp., Redmond, WA, USA) and analyzed using SPSS version 21 (IBM Corp., Armonk, NY, USA), and results were interpreted.

Results

The prevalence of RTI was found to be 50.3%, with the majority (61.3%) of women in the age group of 28-37 years, 52.85% among females living with spouses, and 57.9% from the Hindu community. The prevalence was high among the lower-middle-class and nuclear families. The commonest symptom is vulval itching with 74.09%, and the least is boils with 0.9%. A significant association was noted between RTI and menstrual hygiene practices and socioeconomic status (p < 0.05).

Conclusion

The prevalence was high among rural females, and the main reason behind it was the stigma and the lack of awareness. Health education using various sources should be provided to get rid of these issues.

## Introduction

Reproductive tract infection (RTI) is the infection of the reproductive tract; it is of three types: sexually transmitted infection (STI) such as chlamydia, gonorrhea, chancroid, and human immunodeficiency virus; endogenous infection due to the overgrowth of the normal flora of the reproductive tract; and iatrogenic infection, mainly due to improper procedures such as unsafe abortion and unhygienic delivery practices [1]. The WHO estimates that 80%-90% of the global burden lies in low-middle-income countries (LMICs) [2]. Although reproductive tract infection involves both men and women, it is most common among women, especially in the reproductive age group and specifically the ever-married women. The infection accounts for about 33% in females and 12.3% in males [3]. Every year, thousands of women expire due to or as the sequel of reproductive tract infection [1].

Globally, the prevalence of morbidity among females due to reproductive tract infection accounts for 22%, with the highest prevalence in South Asia and Sub-Saharan Africa with 150 million cases out of 340 million cases [4]. Curable infections such as bacterial vaginosis, gonorrhea, chlamydia, lymphogranuloma venereum, syphilis, trichomoniasis, and chancroid are common compared to incurable infections caused by the human papillomavirus, herpes simplex virus, and human immunodeficiency virus. In India, the annual incidence of reproductive tract infection and sexually transmitted diseases is projected as 5% or approximately 40 million every year [5]. According to the National Family Health Survey 4 report, 89.5% of rural women of the reproductive age group follow good menstrual hygiene, especially the age group of 15-24 years, among which only 15.6% were aware of reproductive tract infection and their spread [6]. The District Level Household Survey 4 (DLHS-4) conducted during 2012-2013 reported that the awareness about reproductive tract infection among the rural population of Tamil Nadu is 8%, and the awareness about the symptoms is 55.7% [7].

The most common presenting complaint of reproductive infection is vaginal discharge, the leading cause of gynecological morbidity [8]. Keeping in mind the complications and sequelae, the prevention, control, and management of reproductive tract infection are given high precedence in national programs such as Reproductive and Child Health II and National AIDS Control Program (NACP-IV) [9]. The key plan of the Reproductive and Child Health II and National AIDS Control Program is the execution of the syndromic management [9]. The syndromic management of reproductive tract infections/sexually transmitted infections is based on the symptoms and signs that are associated with the infection. The main objective of this approach is to identify and treat the syndromes with the blended therapy that covers the contributing organisms. This system is vastly sensitive, and the treatment is also given at the primary care level. The easy flow diagrams in this guideline help health workers in the early detection and treatment of the disease using laboratory analysis. The core drawback of the syndromic management approach is that 87% of the fund is spent on overtreatment [10]. The use of contraceptives also plays a vital role; users of intrauterine contraceptive devices (IUCDs) have a higher risk of developing infection compared with users of barrier contraceptives [11], and the control of reproductive tract infection is a crucial health priority in several countries [12]. Overcrowding also leads to the high prevalence of reproductive tract infections [13].

The Ministry of Health and Family Welfare, Government of India, decided to start the process of conducting DLHS-4 during the year 2012-2013. The questionnaires enclosing details about reproductive tract infection were considered for the study [7].

With this as the backdrop and with women from rural areas themselves being a high-risk factor for reproductive tract infection, this study mainly focused on estimating the prevalence of reproductive tract infection using the syndromic management approach among rural women in the reproductive age group, i.e., 18-49 years, in Kancheepuram District, Tamil Nadu.

## Materials and methods

Study design, period, and area

This study is a community-based cross-sectional study conducted for a period of 14 months from March 2016 to May 2017 in the field practice area of Chettinad Hospital and Research Institute.

Study population

The total population of villages in the field practice area is 39,545, among which 20,480 are males and 19,065 are female. From this female population, those who are less than 18 years old and more than 49 years old (N=14,003) were excluded, and 5,062 females under the reproductive age group were listed; samples are selected for the study.

Inclusion criteria

Ever-married females in the reproductive age group were included.

Exclusion criteria

Antenatal females, postnatal mothers, postmenopausal women, and women with terminal illnesses were excluded from the study.

Sample size

With 24% as average precision [14-16], 95% confidence interval, and 5% absolute error, the sample size calculated using the formula 4pq/d^2^ was 292, and accounting for 15% nonresponse rate, the sample size obtained was 330.

Sampling technique

A multistage random sampling technique was used to select villages, and each village is considered a cluster. The population proportion to size method was used in selecting the samples from each cluster.

Study tool

A structured questionnaire containing sociodemographic factors such as age, educational status, qualification, socioeconomic status, family profile, environmental history, and menstrual and obstetric history; a standard questionnaire consisting of menstrual hygiene and personal hygiene practices; and questions eliciting symptoms of reproductive tract infection, such as vaginal discharge, vulval itching, abdominal pain, low backache, dyspareunia, and post-coital spotting, along with questions describing their pattern of treatment, were used as a study tool.

Data collection

The questionnaires were explained to ever-married females in the reproductive age group and filled out using the interview method.

Statistical analysis

The data collected were entered in Microsoft Excel (Microsoft Corp., Redmond, WA, USA), and statistical analysis was done using the SPSS software version 21 (IBM Corp., Armonk, NY, USA) [17]. The Chi-square test was applied for significance. P-value < 0.05 was considered significant.

Ethical consideration

The study was conducted after obtaining ethical approval from the institutional ethical committee of Chettinad Hospital and Research Institute with IRB number 23/ IHEC/ 3-16. Informed written consent was obtained from the participants.

## Results

The prevalence of reproductive tract infection using the syndromic management approach among the rural women of the reproductive age group in Kancheepuram District was 50.3%. Table 1 shows the distribution of the study participants with the reproductive tract infection based on sociodemographic factors. The prevalence of this infection was high among the young adult females who are 18-27 years old (61.3%). According to literacy status, the prevalence of reproductive tract infection was comparatively found to be high in the graduates (48.2%), and it also increases with the decreasing level of socioeconomic status and higher in the lower-middle class (52.9%). The prevalence of infection was high among females living with spouses (52.8%) and living in nuclear families (53.6%).

**Table 1:** Distribution of RTI among the study participants according to selected sociodemographic characteristics

Variables	Total (N=330)	RTI (N=166)
Age group		
18–27	75	46 (61.3%)
28–37	156	74 (47.3%)
38–49	99	46 (46.4%)
Marital status		
Living with husband	299	158 (52.8%)
Widow	18	5 (27.2%)
Divorce	13	3 (23%)
Religion		
Hindu	233	135 (57.9%)
Christian	66	27 (40.9%)
Muslim	31	4 (12.9%)
Educational status		
Primary	56	23 (41%)
Middle school	32	26 (81.2%)
High school	79	35 (44.3%)
Higher secondary	54	25 (46.2%)
Graduate	58	28 (48.2%)
Illiterate	51	19 (37.2%)
Socioeconomic class		
Upper	9	2 (22.2%)
Upper middle	49	20 (40.8%)
Lower middle	134	71 (52.9%)
Upper lower	103	51 (49.5%)
Lower	35	16 (45.7%)
Type of family		
Nuclear	194	104 (53.6%)
Joint	81	40 (49.3%)
Three generation	55	22 (40%)

The prevalence of infection was high (80%) among females who had poor personal hygiene and menstrual hygiene practices (69.2%). Table 2 shows the prevalence of reproductive tract infection based on personal hygiene practices such as sanitary practice and menstrual hygiene followed.

**Table 2:** Distribution of RTI among the study participants according to hygiene practices

Hygiene practices	Total (N=330)	RTI (N=166)
Sanitary facility		
Good	320	158 (49.3%)
Poor	10	8 (80%)
Menstrual hygiene		
Sanitary napkin	242	106 (43.8%)
Cloth	78	54 (69.2%)
Locally prepared napkin	10	6 (60%)

The most common symptom presented by the study participants was vulval itching with 74.09%, while the least common complaint was boils with 1.8%. Table 3 describes the prevalence of the various symptoms of reproductive tract infection among the study participants.

**Table 3:** Prevalence of the symptoms of reproductive tract infection

Symptoms of reproductive tract infection	Frequency (N=166)	Percentage (%)
Vulval itching	123	74.09
Low backache	120	72.28
Vaginal discharge	105	63.25
Dyspareunia	48	28.91
Lower abdominal pain	36	21.68
Dysmenorrhea	7	04.21
Post-coital bleeding	3	01.80
Boils	3	01.80

A significant association was noted among the age factor and the symptoms of vulval itching and dyspareunia, while educational status had an association significant only with dyspareunia, as an increasing level of education decreases the stigma about the disease. A significant association was noted between socioeconomic status and vulval itching, low backache, and dyspareunia. Table 4 shows the association between the various demographic factors and the various symptoms of reproductive tract infection.

**Table 4:** Association between symptoms of reproductive tract infection and its determinants *Significant at a p-value of 0.05

Demographic characteristics	Reproductive tract infection symptoms						
	Vulval itching (N (%))	Low backache (N (%))	Vaginal discharge (N (%))	Dyspareunia (N (%))	Dysmenorrhea (N (%))	Lower abdominal pain (N (%))	Post-coital bleeding (N (%))
Age (years)							
18–27	36 (29.3)	33 (27.5)	31 (29.5)	15 (31.3)	9 (25)	1 (14.3)	1 (33.3)
28–37	58 (47.2)	55 (45.8)	49 (46.7)	27 (56.3)	16 (44.4)	6 (85.7)	2 (66.7)
38–49	29 (23.6)	32 (26.7)	25 (23.8)	61 (12.5)	11 (30.6)	0	0
Chi-square	6.338	2.671	5.109	8.487	0.164	4.605	1.299
P-value	0.041*	0.263	0.078	0.014*	0.921	0.100	0.52
Education							
Primary	18 (14.6)	19 (15.8)	16 (15.2)	3 (6.3)	4 (11.1)	1 (14.3)	0
Middle school	18 (14.6)	17 (14.2)	11 (10.5)	12 (25)	5 (13.9)	0	0
High school	32 (26)	22 (18.3)	27 (25.7)	5 (10.4)	5 (13.9)	1 (14.3)	0
Higher secondary	14 (11.4)	21 (17.5)	18 (17.1)	14 (29.2)	10 (27.8)	2 (28.6)	1 (33.3)
Graduate	25 (20.3)	19 (15.8)	18 (17.1)	11 (22.9)	5 (13.4)	2 (28.6)	1 (33.3)
Illiterate	16 (13)	22 (18.3)	15 (14.3)	3 (6.3)	0	1 (14.3)	1 (33.3)
Chi-square	10.489	7.990	0.781	31.277	17.201	2.152	3.119
P-value	0.063	0.157	0.978	0.000*	0.206	0.828	0.682
Socioeconomic status							
Upper class	2 (1.6)	3 (2.5)	2 (1.9)	1 (2.1)	1 (2.8)	0	0
Upper middle	10 (8.1)	12 (10)	15 (14.3)	11 (22.9)	8 (22.2)	0	0
Lower middle	51 (41.5)	70 (58.3)	45 (42.9)	10 (20.8)	20 (55.6)	2 (28.6)	0
Upper lower	46 (37.4)	27 (22.5)	37 (35.2)	15 (31.3)	6 (16.7)	4 (57.1)	2 (66.7)
Lower	14 (11.4)	8 (6.7)	6 (5.7)	11 (22.9)	1 (2.8)	1 (14.3)	1 (33.3)
Chi-square	9.384	24.960	4.881	15.982	8.778	3.144	4.555
P-value	0.050*	0.000*	0.300	0.0003*	0.067	0.534	0.348
Marital status							
Living with spouse	107 (87)	112 (93.3)	93 (88.6)	46 (95.8)	36 (100)	7 (100)	3 (100)
Widow	9 (7.3)	5 (4.2)	7 (6.7)	0	0	0	0
Divorce	6 (4.9)	2 (1.7)	4 (3.8)	1 (2.1)	0	0	0
Chi-square	3.607	4.988	2.615	2.022	4.339	0.768	0.325
P-value	0.307	0.173	0.455	0.568	0.227	0.857	0.955

No association was noted between personal hygiene practices and symptoms of reproductive tract infection, while menstrual hygiene practices had a significant association with vaginal discharge and dysmenorrhea. Table 5 shows the association between hygiene practices such as sanitation and menstrual hygiene and various symptoms of reproductive tract infection.

**Table 5:** Association between symptoms of reproductive tract infection and hygiene practices *Significant at a p-value of 0.05

Hygiene practices	Reproductive tract infection symptoms						
	Vulval itching (N (%))	Low backache (N (%))	Vaginal discharge (N (%))	Dyspareunia (N (%))	Dysmenorrhea (N (%))	Lower abdominal pain (N (%))	Post-coital bleeding (N (%))
Sanitary facility							
Good	121 (98.4)	119 (99.2)	103 (98.1)	48 (100)	35 (97.2)	7 (100)	3 (100)
Poor	2 (1.6)	1 (0.8)	2 (1.9)	0	1 (2.8)	0	0
Chi-square	1.316	3.907	.664	1.755	0.009	0.223	0.095
P-value	0.332	0.100	0.512	0.368	1.000	1.000	1.000
Menstrual hygiene practices							
Sanitary napkin	97 (78.9)	92 (76.7)	99 (94.3)	37 (77.1)	35 (97.2)	7 (100)	3 (100)
Cloth	21 (17.1)	26 (21.7)	2 (1.9)	11 (22.9)	0	0	0
Locally prepared napkin	5 (4.1)	2 (1.7)	4 (3.8)	0	1 (2.8)	0	0
Chi-square	5.084	1.752	40.297	1.822	12.704	2.601	1.101
P-value	0.079	0.416	0.000*	0.402	0.002*	0.272	0.577

## Discussion

The findings of our community-based study among ever-married females of the reproductive age group in the rural areas of Kancheepuram District indicated that more than half (50.3%) of them were suffering from either one of the symptoms of RTI. These symptoms were high among the age group of 18-27 years (61.3%), and it was also high among females who were living with their husbands (52.8%) compared to widowed and divorced females. Symptoms were more among the Hindu community (57.9%), those with middle school standard (81.2%), and those with lower-middle-class socioeconomic status (52.9%). The commonest symptom was vulval itching (74.09%), which was high among the age group of 28-37 years; it was 41.5% in the lower-middle class, 73.2% among Hindu females, and 87% among females living with their partners. It was followed by low backache (72.28%), which is high among those who are 28-37 years old (45.8%), in the lower-middle class (58.3%), Hindu (76.7%), and living with spouses (93.3%). Vaginal discharge was at 63.25%, which was high among the age group of 28-37 years (46.7%), females in the lower-middle class (42.9%), those living with spouses (88.6%), and those living in nuclear families (64.8%).

A similar study conducted in Surendranagar District indicated a prevalence rate of 56.5%, and the prevalence was common among the low socioeconomic group. The commonest symptom reported was vaginal discharge with 26.3% [13]. The prevalence of RTI was estimated to be 51.9% in a study conducted in Sirmaur; the frequently occurring symptom was vaginal discharge with 51.9%, and the age group affected was 25-34 years, with a prevalence of 63.6%. There was an increasing trend of prevalence among the illiterate, with a prevalence of 72%, with a frequent complaint of vaginal discharge (51.9%) [17]. A study conducted in Raichur reported a prevalence rate of 58.9%, the most common symptom being vaginal discharge (27%). The prevalence was high among the mid-reproductive age group (25-34 years), and socioeconomic status plays a major role in the prevalence of RTI [18]. The study that was conducted in the Chennai basis areas showed a prevalence of reproductive tract infection of 45.5%, with the common symptom being vaginal discharge (35%) [19]. The epidemiological study conducted in the Bundelkhand region of Uttar Pradesh reported a prevalence of infection of 44.6%, with the common presenting symptom being vaginal discharge (74.2%), followed by vulval itching (35.6%). The prevalence was high among the age group of 25-29 years and those with low socioeconomic status, and there is also an association between literature status and the prevalence of infection. The prevalence was high among cloth users with 43.6% and those using sanitary pads with 31.3% [20]. A community-based study on reproductive tract infection in a district of West Bengal indicated a very low prevalence of 9.85%. The presenting symptom vaginal discharge falls under the high category, with a prevalence of 45%. The prevalence among the age group of 24-29 years was high with 11.39%. Females who reported the symptoms had low socioeconomic status (53.5%), and it is 6.89% among those with higher standards [21]. A cross-sectional study of a rural community in Hooghly District reported the prevalence of reproductive tract infection to be 13.7%. Vaginal discharge is the most common symptom (7.5%) [9].

Limitation of the study

The prevalence of the RTI was estimated only based on the symptoms of the infection presented by the study participants; clinical examination and laboratory investigations were not conducted to confirm any infections.

## Conclusions

In conclusion, RTI was common among females of the reproductive age group in the rural community with high prevalence such that more than half of the study population had either one of the symptoms of reproductive tract infection, and the association was also found to be significant. This is mainly due to several reasons, such as lack of awareness about the symptom of the disease, stigma in using terms such as white discharge, vulval itching, and pain during sexual contact that describe the symptoms associated with the disease, and poor menstrual hygiene and personal hygiene practices.

To overcome all these reasons, females of the reproductive age group in rural areas should be provided with regular health education regarding the symptoms of RTI and should also be motivated to seek proper management for that particular complaint. Health education in the area of menstrual hygiene and personal hygiene should be provided not only to females of the reproductive age group but also to females of the adolescent age group, which can help in reducing the prevalence of reproductive tract infection. Our study also suggests that health education should be mainly imparted to females of low socio-economic class and early adults to overcome the symptoms of RTI, and this might be an immediately feasible method to decrease the burden of the disease in the community. Health education on personal and menstrual hygiene practices using the various study material (IEC) is recommended to decrease the burden of the problem, and it is also most important to involve adolescent females, those both at high school and college, in health education for the betterment of the situation.
